# (±)-1-(1*H*-Benzimidazol-2-yl)ethanol

**DOI:** 10.1107/S1600536808013871

**Published:** 2008-06-07

**Authors:** Rong Xia, Hai-Jun Xu

**Affiliations:** a Ordered Matter Science Research Center, College of Chemistry and Chemical Engineering, Southeast University, Nanjing 210096, People’s Republic of China

## Abstract

The asymmetric unit of the title mol­ecule, C_9_H_10_N_2_O, contains two mol­ecules. The fused benzene and imidazole rings are nearly coplanar, the largest deviations from the mean plane being 0.025 (3) Å at the non-bridgehead imidazole C atom of one mol­ecule and 0.018 (3) Å at one of the bridgehead C atoms in the other mol­ecule. Intermolecular O—H⋯N and N—H⋯O hydrogen bonds result in the formation of a sheet parallel to the (010) plane.

## Related literature

For related literature, see: Allen *et al.* (1987 ▶); Chen & Ruan (2007 ▶); Garuti *et al.* (1999 ▶); Matsuno *et al.* (2000 ▶); Tlahuext *et al.* (2007 ▶).
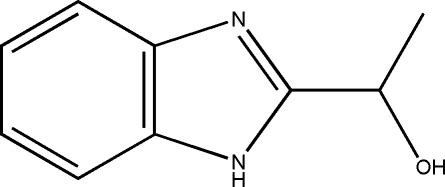


## Experimental

### 

#### Crystal data

C_9_H_10_N_2_O
*M* *_r_* = 162.19
Orthorhombic, 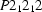

*a* = 13.734 (3) Å
*b* = 15.376 (3) Å
*c* = 7.9163 (16) Å
*V* = 1671.7 (6) Å^3^
*Z* = 8
Mo *K*α radiation
μ = 0.09 mm^−1^
*T* = 293 (2) K
0.20 × 0.18 × 0.05 mm

#### Data collection

Rigaku Mercury2 diffractometer
Absorption correction: multi-scan (*CrystalClear*; Rigaku, 2005 ▶) *T*_min_ = 0.912, *T*_max_ = 1.00 (expected range = 0.908–0.996)
17487 measured reflections
2199 independent reflections
1380 reflections with *I* > 2σ(*I*)
*R*_int_ = 0.115

#### Refinement

*R*[*F* ^2^ > 2σ(*F* ^2^)] = 0.059
*wR*(*F* ^2^) = 0.123
*S* = 1.07
2199 reflections
221 parameters
H-atom parameters constrained
Δρ_max_ = 0.17 e Å^−3^
Δρ_min_ = −0.18 e Å^−3^

### 

Data collection: *CrystalClear* (Rigaku, 2005 ▶); cell refinement: *CrystalClear*; data reduction: *CrystalClear*; program(s) used to solve structure: *SHELXS97* (Sheldrick, 2008 ▶); program(s) used to refine structure: *SHELXL97* (Sheldrick, 2008 ▶); molecular graphics: *ORTEPIII* (Burnett & Johnson, 1996 ▶), *ORTEP-3* (Farrugia, 1997 ▶) and *PLATON* (Spek, 2003 ▶); software used to prepare material for publication: *SHELXL97*.

## Supplementary Material

Crystal structure: contains datablocks I, global. DOI: 10.1107/S1600536808013871/dn2341sup1.cif

Structure factors: contains datablocks I. DOI: 10.1107/S1600536808013871/dn2341Isup2.hkl

Additional supplementary materials:  crystallographic information; 3D view; checkCIF report

## Figures and Tables

**Table 1:** Hydrogen-bond geometry (Å, °)

*D*—H⋯*A*	*D*—H	H⋯*A*	*D*⋯*A*	*D*—H⋯*A*
O1—H1⋯N3	0.82	1.91	2.713 (4)	165
N4—H4⋯O1^i^	0.86	1.97	2.828 (4)	178
O2—H2⋯N2^ii^	0.82	1.93	2.743 (4)	170
N1—H1*A*⋯O2^iii^	0.86	1.93	2.751 (4)	160

Symmetry codes: (i) 
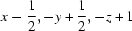
; (ii) 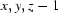; (iii) 
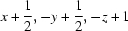
.

**Fig. 1.**
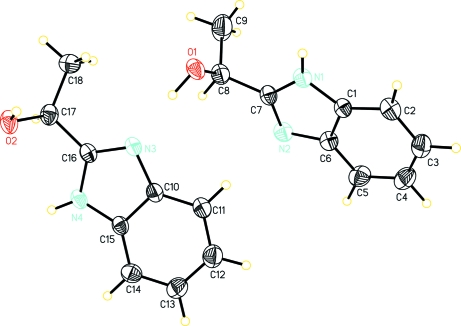
A view of the title compound with the atom-labelling scheme. Displacement ellipsoids are drawn at the 30% probability level. Hydrogen bond is shown as dashed line. H atoms are represented as small spheres of arbitrary radii.

**Fig. 2.**
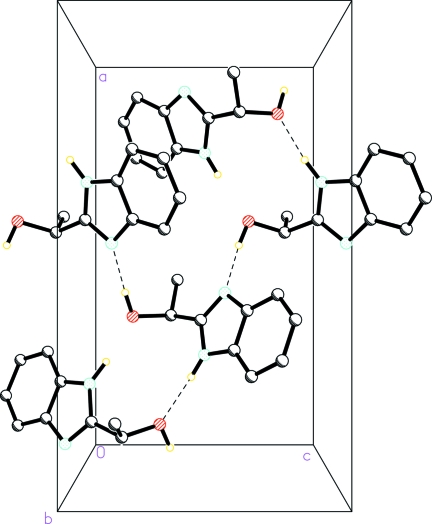
Partial packing view showing the hydrogen bonds network. Hydrogen bonds are shown as dashed lines. H atoms not involved in hydrogen bondings have been omitted for clarity. [Symmetry codes: (i) x-1/2, -y+1/2, -z+1; (ii) x, y, z-1; (iii) x+1/2, -y+1/2, -z+1 ]

## supplementary crystallographic information

### Comment

Imidazole and benzimidazole derivatives are important heteroaromatic compounds and have attracted considerable attention because of good biological and pharmaceutical activities (Matsuno *et al.*, 2000; Garuti *et al.*, 1999). These compounds also play an important role in the development of coordination chemistry. Many derivatives of benzimidazole have been prepared and their complexes have been studied (Tlahuext *et al.*, 2007; Chen & Ruan, 2007). In this paper, we report the crystal structure of the title compound.

There are two crystallographically independent molecules, A and B, linked by a O-H···N hydrogen bond in the asymmetric unit . The bond lengths and angles in A and B are within normal ranges (Allen *et al.*, 1987). The two fused benzene and imidazole rings are nearly planar with the largest deviations from the mean plane being 0.025 (3) Å at C7 and 0.018 (3) Å at C10 . These two fused rings make a dihedral angle of 35.01 (9)°.

The molecules are further connected through O-H···N and N-H···O hydrogen bond buiding up a two dimmensional network which is parallel to the (0 1 0) plane (Table 1, Fig. 2).

Only the relative absolute configuration could be determined, the C8 and C17 have the same absolute configuration (S,S) or (R,R). The (S,S) configuration is represented in Fig. 1.

### Experimental

All chemicals were obtained from commercial sources and used directly without further purification. Benzene-1, 2-diamine (2.16 g, 20 mmol) was dissolved in hydrochloric acid (25 mL, 4 *M*) at 100°C, and ethyl 2-hydroxypropanoate (2.48 g, 21 mmol) was added to the solution. The mixture were then heated to reflux for 7 h at 115°C. After cooling to room temperature, the product was divided by neutralizing the mixture solution using NaOH to make the pH 7–9. Solid product was collected by filtration and the yield was 80%. ^1^H-NMR(CDCl_3_, 300 MHz): δ1.72 (d, 3 H), 5.22 (q, 1 H), 7.47(m, 1 H), 7.58 (m, 2 H), 7.81 (m, 1 H). Esi-MS: calcd for C_14_H_9_N_2_O–H *m*/*z *161.19, found 161.18. Deep-red single crystals of the title compound suitable for X-ray diffraction analysis were obtained from methanol solution by slow evaporation after a week.

### Refinement

All H atoms attached to C, O and N atom were fixed geometrically and treated as riding with C—H = 0.98 Å (methine), 0.96 Å (methyl or 0.93 Å (aromatic), O—H = 0.82 Å and N—H = 0.86 Å with U_iso_(H) = 1.2U_eq_(C,N) or U_iso_(H) = 1.5U_eq_(C_methyl_, O).

In the absence of significant anomalous scattering, the absolute configuration could not be reliably determined and then the Friedel pairs were merged and any references to the Flack parameter were removed.

### Crystal data

**Table:**

C_9_H_10_N_2_O	*F*_000_ = 688
*M**_r_* = 162.19	*D*_x_ = 1.289 Mg m^−^^3^
Orthorhombic, *P*2_1_2_1_2	Mo *K*α radiation λ = 0.71073 Å
Hall symbol: P 2 2ab	Cell parameters from 10749 reflections
*a* = 13.734 (3) Å	θ = 2.4–28.0º
*b* = 15.376 (3) Å	µ = 0.09 mm^−^^1^
*c* = 7.9163 (16) Å	*T* = 293 (2) K
*V* = 1671.7 (6) Å^3^	Block, red
*Z* = 8	0.20 × 0.18 × 0.05 mm

### Data collection

**Table:**

Rigaku Mercury2 diffractometer	2199 independent reflections
Radiation source: fine-focus sealed tube	1380 reflections with *I* > 2σ(*I*)
Monochromator: graphite	*R*_int_ = 0.115
Detector resolution: 13.6612 pixels mm^-1^	θ_max_ = 27.5º
*T* = 293(2) K	θ_min_ = 3.0º
ω scans	*h* = −17→17
Absorption correction: multi-scan(CrystalClear; Rigaku, 2005)	*k* = −19→19
*T*_min_ = 0.912, *T*_max_ = 1.00	*l* = −10→10
17487 measured reflections	

### Refinement

**Table:**

Refinement on *F*^2^	Secondary atom site location: difference Fourier map
Least-squares matrix: full	Hydrogen site location: inferred from neighbouring sites
*R*[*F*^2^ > 2σ(*F*^2^)] = 0.059	H-atom parameters constrained
*wR*(*F*^2^) = 0.123	*w* = 1/[σ^2^(*F*_o_^2^) + (0.0395*P*)^2^ + 0.22*P*] where *P* = (*F*_o_^2^ + 2*F*_c_^2^)/3
*S* = 1.07	(Δ/σ)_max_ < 0.001
2199 reflections	Δρ_max_ = 0.17 e Å^−^^3^
221 parameters	Δρ_min_ = −0.18 e Å^−^^3^
Primary atom site location: structure-invariant direct methods	Extinction correction: none

### Special details

**Table:**

Geometry. All esds (except the esd in the dihedral angle between two l.s. planes) are estimated using the full covariance matrix. The cell esds are taken into account individually in the estimation of esds in distances, angles and torsion angles; correlations between esds in cell parameters are only used when they are defined by crystal symmetry. An approximate (isotropic) treatment of cell esds is used for estimating esds involving l.s. planes.
Refinement. Refinement of *F*^2^ against ALL reflections. The weighted *R*-factor *wR* and goodness of fit *S* are based on *F*^2^, conventional *R*-factors *R* are based on *F*, with *F* set to zero for negative *F*^2^. The threshold expression of *F*^2^ > σ(*F*^2^) is used only for calculating *R*-factors(gt) *etc*. and is not relevant to the choice of reflections for refinement. *R*-factors based on *F*^2^ are statistically about twice as large as those based on *F*, and *R*- factors based on ALL data will be even larger.

### Fractional atomic coordinates and isotropic or equivalent isotropic displacement parameters (Å^2^)

**Table:**

	*x*	*y*	*z*	*U*_iso_*/*U*_eq_	
C1	0.6823 (3)	0.3355 (2)	1.1332 (4)	0.0383 (9)	
C2	0.7541 (3)	0.3880 (2)	1.1995 (5)	0.0496 (10)	
H2A	0.8168	0.3885	1.1551	0.060*	
C3	0.7292 (3)	0.4388 (3)	1.3323 (5)	0.0605 (12)	
H3	0.7760	0.4750	1.3801	0.073*	
C4	0.6362 (4)	0.4384 (3)	1.3984 (6)	0.0652 (13)	
H4A	0.6219	0.4740	1.4900	0.078*	
C5	0.5647 (3)	0.3868 (3)	1.3328 (6)	0.0622 (12)	
H5	0.5021	0.3872	1.3774	0.075*	
C6	0.5887 (3)	0.3340 (2)	1.1975 (5)	0.0427 (9)	
C7	0.5899 (3)	0.2474 (2)	0.9864 (5)	0.0428 (9)	
C8	0.5593 (3)	0.1874 (3)	0.8498 (5)	0.0520 (11)	
H8	0.4882	0.1823	0.8527	0.062*	
C9	0.6018 (4)	0.0985 (3)	0.8690 (6)	0.0919 (18)	
H9A	0.5832	0.0632	0.7743	0.138*	
H9B	0.5781	0.0725	0.9713	0.138*	
H9C	0.6715	0.1027	0.8737	0.138*	
C10	0.3684 (3)	0.3451 (2)	0.6778 (5)	0.0423 (9)	
C11	0.4085 (3)	0.3972 (3)	0.8015 (5)	0.0563 (11)	
H11	0.4721	0.3888	0.8385	0.068*	
C12	0.3517 (3)	0.4614 (3)	0.8676 (5)	0.0614 (12)	
H12	0.3768	0.4974	0.9514	0.074*	
C13	0.2571 (3)	0.4740 (3)	0.8120 (5)	0.0576 (11)	
H13	0.2203	0.5186	0.8591	0.069*	
C14	0.2166 (3)	0.4234 (2)	0.6914 (5)	0.0511 (10)	
H14	0.1527	0.4317	0.6561	0.061*	
C15	0.2740 (3)	0.3592 (2)	0.6231 (4)	0.0401 (9)	
C16	0.3419 (2)	0.2520 (2)	0.4833 (4)	0.0380 (8)	
C17	0.3509 (3)	0.1825 (2)	0.3527 (4)	0.0463 (10)	
H17	0.2934	0.1450	0.3628	0.056*	
C18	0.4389 (3)	0.1262 (3)	0.3790 (6)	0.0581 (11)	
H18A	0.4414	0.0826	0.2924	0.087*	
H18B	0.4349	0.0986	0.4876	0.087*	
H18C	0.4965	0.1614	0.3739	0.087*	
N1	0.68087 (19)	0.27939 (19)	0.9990 (3)	0.0451 (8)	
H1A	0.7292	0.2669	0.9345	0.054*	
N2	0.5316 (2)	0.2773 (2)	1.1042 (4)	0.0486 (8)	
N3	0.4093 (2)	0.27755 (19)	0.5867 (4)	0.0443 (8)	
N4	0.25902 (19)	0.29851 (18)	0.4998 (4)	0.0425 (7)	
H4	0.2063	0.2912	0.4429	0.051*	
O1	0.58658 (16)	0.2215 (2)	0.6915 (3)	0.0532 (7)	
H1	0.5385	0.2415	0.6440	0.080*	
O2	0.34919 (16)	0.21938 (19)	0.1892 (3)	0.0546 (7)	
H2	0.4018	0.2426	0.1697	0.082*	

### Atomic displacement parameters (Å^2^)

**Table:**

	*U*^11^	*U*^22^	*U*^33^	*U*^12^	*U*^13^	*U*^23^
C1	0.041 (2)	0.046 (2)	0.0280 (19)	−0.0019 (17)	0.0020 (17)	0.0067 (17)
C2	0.042 (2)	0.060 (3)	0.047 (2)	−0.0057 (19)	0.000 (2)	0.008 (2)
C3	0.065 (3)	0.065 (3)	0.052 (3)	−0.006 (2)	−0.009 (3)	−0.005 (2)
C4	0.086 (4)	0.060 (3)	0.050 (3)	0.001 (3)	0.002 (3)	−0.009 (2)
C5	0.059 (3)	0.073 (3)	0.055 (3)	0.000 (2)	0.018 (2)	−0.008 (3)
C6	0.041 (2)	0.051 (2)	0.036 (2)	−0.0025 (18)	0.0054 (19)	0.0037 (19)
C7	0.039 (2)	0.052 (2)	0.037 (2)	−0.0042 (19)	−0.0017 (19)	0.0043 (18)
C8	0.047 (2)	0.063 (3)	0.046 (2)	−0.0012 (19)	−0.002 (2)	0.000 (2)
C9	0.135 (5)	0.062 (3)	0.078 (4)	0.008 (3)	−0.040 (4)	−0.005 (3)
C10	0.042 (2)	0.044 (2)	0.040 (2)	−0.0017 (17)	−0.007 (2)	0.0045 (18)
C11	0.057 (3)	0.057 (3)	0.055 (3)	0.005 (2)	−0.015 (2)	−0.003 (2)
C12	0.076 (3)	0.057 (3)	0.051 (3)	−0.002 (2)	−0.009 (3)	−0.003 (2)
C13	0.064 (3)	0.056 (3)	0.053 (3)	0.003 (2)	0.006 (3)	−0.006 (2)
C14	0.043 (2)	0.054 (3)	0.057 (3)	0.0029 (19)	0.005 (2)	0.003 (2)
C15	0.040 (2)	0.048 (2)	0.0323 (19)	0.0002 (18)	−0.0009 (18)	0.0053 (18)
C16	0.0317 (19)	0.042 (2)	0.040 (2)	−0.0067 (16)	−0.0017 (18)	0.0069 (17)
C17	0.041 (2)	0.055 (2)	0.042 (2)	−0.0082 (18)	−0.0007 (19)	−0.0018 (19)
C18	0.053 (2)	0.056 (3)	0.065 (3)	0.009 (2)	−0.005 (2)	−0.005 (2)
N1	0.0335 (16)	0.066 (2)	0.0355 (16)	0.0029 (15)	0.0070 (15)	−0.0025 (17)
N2	0.0388 (17)	0.063 (2)	0.0443 (17)	−0.0022 (16)	0.0071 (15)	0.0033 (17)
N3	0.0394 (17)	0.048 (2)	0.0453 (17)	0.0020 (16)	−0.0106 (16)	−0.0019 (16)
N4	0.0292 (15)	0.0576 (19)	0.0407 (16)	0.0002 (15)	−0.0056 (15)	0.0033 (17)
O1	0.0353 (14)	0.088 (2)	0.0368 (14)	0.0083 (14)	−0.0012 (13)	0.0011 (16)
O2	0.0335 (14)	0.089 (2)	0.0413 (14)	−0.0056 (14)	−0.0032 (13)	−0.0031 (15)

### Geometric parameters (Å, °)

**Table:**

C1—N1	1.368 (4)	C10—C15	1.385 (5)
C1—C2	1.379 (5)	C11—C12	1.364 (5)
C1—C6	1.383 (5)	C11—H11	0.9300
C2—C3	1.354 (5)	C12—C13	1.385 (5)
C2—H2A	0.9300	C12—H12	0.9300
C3—C4	1.381 (6)	C13—C14	1.352 (5)
C3—H3	0.9300	C13—H13	0.9300
C4—C5	1.364 (6)	C14—C15	1.374 (5)
C4—H4A	0.9300	C14—H14	0.9300
C5—C6	1.384 (5)	C15—N4	1.366 (4)
C5—H5	0.9300	C16—N3	1.297 (4)
C6—N2	1.386 (5)	C16—N4	1.350 (4)
C7—N2	1.312 (4)	C16—C17	1.492 (5)
C7—N1	1.347 (4)	C17—O2	1.413 (4)
C7—C8	1.482 (5)	C17—C18	1.501 (5)
C8—O1	1.410 (4)	C17—H17	0.9800
C8—C9	1.494 (6)	C18—H18A	0.9600
C8—H8	0.9800	C18—H18B	0.9600
C9—H9A	0.9600	C18—H18C	0.9600
C9—H9B	0.9600	N1—H1A	0.8600
C9—H9C	0.9600	N4—H4	0.8600
C10—C11	1.379 (5)	O1—H1	0.8200
C10—N3	1.384 (4)	O2—H2	0.8200
			
N1—C1—C2	132.5 (3)	C10—C11—H11	121.2
N1—C1—C6	105.2 (3)	C11—C12—C13	121.1 (4)
C2—C1—C6	122.3 (3)	C11—C12—H12	119.5
C3—C2—C1	116.9 (4)	C13—C12—H12	119.5
C3—C2—H2A	121.5	C14—C13—C12	122.0 (4)
C1—C2—H2A	121.5	C14—C13—H13	119.0
C2—C3—C4	121.7 (4)	C12—C13—H13	119.0
C2—C3—H3	119.1	C13—C14—C15	117.1 (4)
C4—C3—H3	119.1	C13—C14—H14	121.5
C5—C4—C3	121.6 (4)	C15—C14—H14	121.5
C5—C4—H4A	119.2	N4—C15—C14	133.3 (4)
C3—C4—H4A	119.2	N4—C15—C10	105.0 (3)
C4—C5—C6	117.6 (4)	C14—C15—C10	121.8 (4)
C4—C5—H5	121.2	N3—C16—N4	112.4 (3)
C6—C5—H5	121.2	N3—C16—C17	126.5 (3)
C1—C6—C5	119.8 (4)	N4—C16—C17	121.1 (3)
C1—C6—N2	109.9 (3)	O2—C17—C16	110.2 (3)
C5—C6—N2	130.3 (4)	O2—C17—C18	111.8 (3)
N2—C7—N1	112.6 (3)	C16—C17—C18	112.6 (3)
N2—C7—C8	124.3 (3)	O2—C17—H17	107.3
N1—C7—C8	123.0 (3)	C16—C17—H17	107.3
O1—C8—C7	110.0 (3)	C18—C17—H17	107.3
O1—C8—C9	109.1 (4)	C17—C18—H18A	109.5
C7—C8—C9	112.6 (3)	C17—C18—H18B	109.5
O1—C8—H8	108.4	H18A—C18—H18B	109.5
C7—C8—H8	108.4	C17—C18—H18C	109.5
C9—C8—H8	108.4	H18A—C18—H18C	109.5
C8—C9—H9A	109.5	H18B—C18—H18C	109.5
C8—C9—H9B	109.5	C7—N1—C1	107.5 (3)
H9A—C9—H9B	109.5	C7—N1—H1A	126.2
C8—C9—H9C	109.5	C1—N1—H1A	126.2
H9A—C9—H9C	109.5	C7—N2—C6	104.7 (3)
H9B—C9—H9C	109.5	C16—N3—C10	105.4 (3)
C11—C10—N3	130.1 (3)	C16—N4—C15	107.7 (3)
C11—C10—C15	120.4 (4)	C16—N4—H4	126.2
N3—C10—C15	109.5 (3)	C15—N4—H4	126.2
C12—C11—C10	117.6 (4)	C8—O1—H1	109.5
C12—C11—H11	121.2	C17—O2—H2	109.5

### Hydrogen-bond geometry (Å, °)

**Table:**

*D*—H···*A*	*D*—H	H···*A*	*D*···*A*	*D*—H···*A*
O1—H1···N3	0.82	1.91	2.713 (4)	165
N4—H4···O1^i^	0.86	1.97	2.828 (4)	178
O2—H2···N2^ii^	0.82	1.93	2.743 (4)	170
N1—H1A···O2^iii^	0.86	1.93	2.751 (4)	160

Symmetry codes: (i) *x*−1/2, −*y*+1/2, −*z*+1; (ii) *x*, *y*, *z*−1; (iii) *x*+1/2, −*y*+1/2, −*z*+1.
